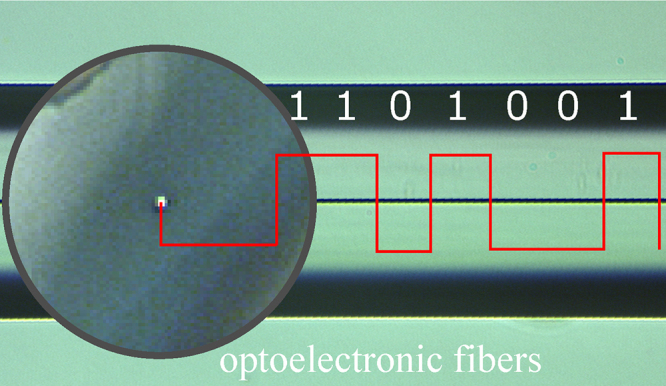# Recent progress of semiconductor optoelectronic fibers

**DOI:** 10.1007/s12200-021-1226-0

**Published:** 2021-06-28

**Authors:** Hei Chit Leo Tsui, Noel Healy

**Affiliations:** Emerging Technologies and Materials Group, School of Mathematics, Statistics and Physics, Newcastle University, Newcastle, NE1 7RU UK

**Keywords:** optical fibers, semiconductor photonics, nonlinear optics

## Abstract

Semiconductor optoelectronic fiber technology has seen rapid development in recent years thanks to advancements in fabrication and post-processing techniques. Integrating the optical and electronic functionality of semiconductor materials into a fiber geometry has opened up many possibilities, such as in-fiber frequency generation, signal modulation, photodetection, and solar energy harvesting. This review provides an overview of the state-of-the-art in semiconductor optoelectronic fibers, including fabrication and post-processing methods, materials and their optical properties. The applications in nonlinear optics, optical-electrical conversion, lasers and multimaterial functional fibers will also be highlighted.

## 1 Introduction

The optical fiber has truly revolutionized the way in which we communicate and share information. Its advent has enabled the high-speed, long-distance, information sharing age that has become the cornerstone of modern society. The first practical demonstration of low-loss light transmission was undertaken using the now ubiquitous silica optical fiber and this remains the major fiber-type for most applications. Silica, for all its strengths, such as, ultra low-loss and natural abundance, does have limitations in terms of the functionality that it provides. As a result, the silica optical fiber is predominately used as a passive carrier of information with signal processing and light generation undertaken elsewhere. Consequently, scientists have been striving to increase the functionality of the optical fiber for decades. The first big breakthrough being the doping of silica optical fibers with rare-earth dopants such as erbium. This technology led to the fiber optical amplifier and the fiber laser, further extending the reach of the silica fiber platform [1–7]. However, the material's limitations, such as the low-solubility of rare-earth dopants, low nonlinear optical coefficient, poor electronic properties and a limited transparency window have prompted numerous investigations into other material systems for next generation fiber platforms [8–11].

One approach is to replace silica with alternative types of glass, which can possess a transparency window extending into the mid-infrared (mid-IR), have higher solubility of rare-earth ions and/or high optical nonlinearities [12–17]. For example, phosphate glasses can accommodate a high concentration of rare-earth ions (up to $10^{21}$ $cm^{-3}$, 50 times higher than silica) because of their open and disordered matrix structure [18]. Furthermore, chalcogenide and fluoride glasses have low phonon energies, which makes them particularly good hosts for rare-earth dopants when being used for efficient mid-IR radiative processes [19,20]. They can also have wide transparent windows from 1 to 22 μm, depending on the composition [21]. Chalcogenide glasses are great candidates for nonlinear optics applications as they possess strong third order nonlinear properties, for instance a nonlinear refractive index ($n_2$) of $2.3 \times 10^{-17}$ $m^2/W$ was reported for $As_{40}Se_{60}$, which is nearly 1000 times higher than that of silica [22,23]. However, owing to their relatively weak chemical bonds, these glass materials usually suffer from poor mechanical properties, low chemical stability, low glass transition temperature and undergo thermal degradation when working with high optical power [18,20,21,24,25].

In recent years, the semiconducting optoelectronic fiber has emerged as a strong candidate to complement the highly successful, but predominantly passive functionality, of the silica optical fiber platform. The semiconductor optoelectronic fiber can be defined as an optical fiber-based platform that includes one or more semiconducting elements in the light's optical path, for example, in the core or in the cladding. This format permits one to leverage the optoelectronic properties of semiconductors, including

Topical Collection: Recent Advances in Functional Fibers many of the technologically important group IV elements and III-V/II-VI compounds. Furthermore, these materials open up extended transmission windows and their high refractive indices and optical nonlinearities permit very efficient fiberized all-optical signal processing and frequency generation. The technology holds the potential to build high efficiency in-fiber optical systems, with light generation, modulation and detection in one fiber, while simultaneously opening up new transmission windows for communications and sensing [10,26–29]. One exciting development is the potential for the application of semiconductor optoelectronic fibers for wearable technologies. With micro-scale thickness and flexible nature, functional fibers can easily be woven into fabrics and textiles [30,31]. Large flexible structures with embedded optoelectronic fibers have shown the potential for novel applications in textiles. For example, positional sensitive light detection and lensless imaging [32,33]. Other promising applications being thermoelectric fibers, temperature monitoring and controlling textiles as well as light-weight wearable energy harvesting devices [34–36].

This review will begin with an introduction to the fabrication and post-processing methods for semiconductor core fibers. Then, a selection of materials and their optical properties will be discussed. Finally, the emerging applications of semiconductor optoelectronic fibers will be presented.

## 2 Fiber fabrication techniques

The two major techniques for producing semiconductor optoelectronic fibers are high pressure assisted deposition and molten core drawing. These fabrication methods are complementary, and their pros and cons are summarized in this section.

### 2.1 Pressure assisted deposition

Polycrystalline silicon-core fiber was first demonstrated by high pressure chemical vapor deposition (HPCVD) in 2006 [37]. In that experiment, silicon was grown within the pore of a microstructured silica capillary, which was used as a reaction chamber for deposition and a cladding layer for the waveguide, see Fig. 1(a) for a schematic of the process. The high pressure is necessary to transport the precursors through the capillary where it is thermally decomposed at the desired deposition site. The smooth surface of the capillary pore can also help produce an atomically smooth surface on the crystal deposit, which is crucial to minimise scattering loss due to surface roughness [38].

The major advantage of HPCVD is that the deposition temperature can be tailored for producing amorphous or crystalline materials as well as a wide range of materials, for instance II-VI and III-V semiconductors. HPCVD also allows crystals to form within a sub-micron capillary diameter, which is essential for single-mode transmission. Another unique capability of the HPCVD process is the ability to permit the deposition of multiple materials in concentric layer structures, thus, enabling the creation of heterostructures for optoelectronic and photonic applications. Furthermore, the low processing temperatures and the nature of the CVD process allows for materials that sublimate, and cannot be easily made molten, to be deposited. However, the slow deposition rate, relatively short fiber lengths, and the difficulty of growing large single crystals are the drawbacks of HPCVD [10,28,39,40].

An alternative to HPCVD is the pressure assisted melt filling (PAMF) method of fiber fabrication [41]. A schematic of the process is shown in Fig. 1(b), in which, molten material is forced into the capillary under high pressure and, in contrast to HPCVD, long cm scale lengths of fiber can be produced in short time-scales (hours). The first demonstration of PAMF was used to produce germanium cladding inclusions in microstructured optical fiber but the approach has been extended to other materials, including glasses and metals. One interesting example being the use of gold to introduce plasmonic functionality into a microstructured optical fiber. Remarkably, the team managed to melt and force gold into pore sizes of just 120 nm diameter, demonstrating the extremely confined spaces that the process can accommodate [42]. The limiting factor for this process is the choice of core material. The core material must have a melting temperature that is lower than the softening temperature of the cladding material, so for example, this precludes the deposition of silicon into a silica capillary as, in this case, the semiconductor has a melting temperature of 1414°C and silica a softening temperature of 1200°C. A further limitation, is the lack of control of the cooling process. This typically renders the resultant core material with poor crystallinity. However, this can be overcome by post-processing, as demonstrated by Chen et al. [43].

### 2.2 Drawing techniques

The molten core drawing (MCD) method is adapted from the powder-in-tube technique, which was originally developed for drawing fiber with core materials that are unstable in standard optical fiber drawing conditions [44,45]. Generally, a glass tube, acting as both a crucible and the fiber's cladding, is filled with the desired semiconductor core material (in the form of powder, a rod, or both). This preform is then heated to above both the cladding glass transition and semiconductor melting temperatures. The fiber is then drawn using the same drawing techniques as is used for standard glass fibers (Fig. 1(c)). As the drawn fiber cools, the molten material becomes a polycrystalline core and the glass tube becomes the cladding [46]. A distinct benefit of MCD is that it

**Fig. 1** Schematics of the various semiconductor optoelectronic fiber fabrication processes. (a) HPCVD method. (b) PAMF method. (c) MCD method allows kilometer-long fiber production with centimeter-long single crystal grains, which is important for achieving low-loss optical transmission. Also, the fabrication process is relatively simple compared to HPCVD. However, due to its high processing temperature, oxygen or other elements can diffuse from the cladding to contaminate and create defects in the core [45,47]. Some solutions to this have been developed, including adding an alkaline oxide modifier at the core-cladding interface which can limit the diffusion and also relieve interfacial stress that originates from the thermal expansion mismatch between the core and cladding material [48].

*In situ* chemical reactions are often viewed as problematic for the MCD process, however, these can be used advantageously. One exciting example is the production of Si core fiber from a preform consisting of just a silica cladding and an aluminum core [49]. During the thermal drawing process, the aluminum acts to reduce the silica to produce silicon, which diffuses into the core forming a large domain of phase-separated molten silicon. A large portion of the drawn fiber then consists of a silica cladding and a silicon core. Another example of *in situ* chemistry via fiber drawing demonstrated the production of ZnSe [50]. A preform of alternating layers of elemental zinc- and selenium-rich films were drawn into meters of fiber at temperatures well below the melting temperature of both Zn or ZnSe. This is a remarkable result as the drawing of bulk ZnSe has not yet been achieved owing to it sublimating at elevated temperature. The authors used the resulting fiber to demonstrate rectifying junction functionality, further signaling the versatility of the method. This body of work demonstrates the potential power of *in situ* chemistry in the thermal drawing of optoelectronic fibers.

## 2.3 Post-processing

As the core materials are mostly in an amorphous or polycrystalline state in the as-produced fibers, post-processing is often necessary to improve the material quality and/or optimise the fiber geometry in order to fully exploit the potential of the semiconductor optoelectronic fibers. All post-processing techniques are dependent on heating the sample either via thermal annealing or melt-recrystallization.

### 2.3.1 Thermal annealing

Annealing is an engineering method for controlling the materials properties through crystal growth. This process does not require that the material is melted and recrystallized, but rather a controlled nucleation and growth of crystals during which defects are reduced and grain sizes are increased. Thermal annealing also holds the potential to grow crystals in preferred orientations, which can be of particular importance for some applications such as nonlinear optics. There are two main annealing processes that have been employed on semiconductor optoelectronic fibers; thermal annealing and rapid photo-thermal annealing [51,52]. In general, the annealing process requires holding the material at high temperatures for sustained periods of time. Both thermal and photothermal annealing have been developed for the silicon fiber platform. The thermal annealing process has predominantly been used to convert amorphous silicon core material, produced via the HPCVD process, into crystalline material. A two-step process has proven to be effective for growing large crystal grains. The first step involves a 3-day low temperature (530°C) anneal to control/limit the number of nucleation sites followed by a second step for rapidly growing these crystallites; typically a 10-min hold at an elevated temperature of 1300°C. The resultant fibers demonstrated a significant lower dislocation density and optical loss than the pre-annealed fiber [53]. In this instance the crystals were limited to sizes of 100 μm.

Rapid-photothermal annealing is a technique that mimics the same process in the semiconductor electronics industry. In contrast to the conventional furnace processing and rapid thermal processing, which rely on thermal energy and photon energy in the visible range, the Xenon lamp used in this method provides light in the ultraviolet and vacuum ultraviolet region. It is believed that this extra optical energy leads to additional electron excitations and reduces the defect formation energy in the crystal. Via this process, the inhomogeneous dislocations were reduced and a homogenous 10 mm long silicon single crystal was achieved, which was double the length of the as-drawn sample [54].

#### 2.3.2 Tapering

Tapering is a technique for reducing the cross-sectional diameter of optical fibers and the process involves heating a section of the fiber while pulling both ends in opposing directions. The predominant concerns when production tapered optoelectronic silicon fibers is the shape of the taper and the drawing stability [55]. To strengthen the fiber and ensure the integrity, Suhailin et al. added a sleeve capillary as a protective layer prior to tapering (Fig. 2(a)) and demonstrated that small core (1 μm) polycrystalline silicon fiber with low loss (3 dB/cm) can be achieved. This novel technique not only improves the crystallinity of the core but also reduces the length of fiber required for the tapering process, thus opening up tapering to fibers produced via HPCVD and PAMF [56,57].

#### 2.3.3 Laser processing

Laser processing of semiconductor optoelectronic fibers is a very versatile approach that can be used to modify the semiconductor material in a number of ways, for example, its degree of crystallinity, its composition, and its optoelectronic properties. In comparison to the more traditional annealing approaches, the semiconductor is heated locally to a molten state and then recrystallized in a highly controlled manner as it is scanned through the focal point of the laser; a schematic is shown in Fig. 2(b). The rate of cooling can be controlled by pulsing the laser but is typically done be altering the focal parameters and the scan speed. The high degree of control over the processing parameters, such as temperature, cooling rate, and localization has meant that the process has led to important advances in the field of semiconductor optoelectronic fibers; including significantly reduced losses, tuned material properties, and compositional microstructuring. While all tapering processes require that the core and cladding materials are heated without discrimination, laser processing can be selective in that the wavelength can be chosen to heat just the core or just the cladding. This has led to there being two dominant heating techniques in the field. The first approach to be demonstrated was the heating and melting of the core by direct optical absorption of the laser beam. A laser source that is transmissible through the cladding material but strongly absorbed by the core via electronic absorption is selected, i.e., a laser with photon energy greater than the semiconductor's electronic bandgap. Using this approach large single crystal core fibers have been produced in both silicon and germanium. However, the strong thermal isolation of the heated core material means that a large strain can be imparted to the core material. The level of strain can be controlled via scan speed and can be harnessed to control the material's

**Fig. 2** (a) Schematic of the tapering process with over-sleeve fiber. (b) Schematic of the laser recrystallization process electronic bandgap. The second approach melts the semiconductor via thermally conducted heat from the cladding. In this instance, the laser is selected so that it is absorbed by the cladding material; for silica-clad fibers, the source is typically a $CO_2$ laser. Using this method, strain can be minimized and single crystals of cm lengths and high aspect ratios ($10^4$) can be grown [58–60]. Finally, both approaches can be undertaken simultaneously in a single process step with energy being absorbed by both the core and cladding.

The high localization of the laser treatment has opened up other opportunities. For example, Coucheron et al. showed that laser processing can help overcome the composition segregation issue in in SiGe core fiber due to the non-equilibrium solidification while graded-index SiGe core fibers have also been realized [61,62]. Fokine et al. demonstrated a Bragg grating with a spacing of 20 μm on fiber by introducing strain within the cladding and at the interface between the glass and the core using a femtosecond laser [63].

## 3 Core materials

The semiconductor optoelectronics fiber platform has proven to be very versatile and fiber materials systems have emerged in the last decade. Each material system offers different functionality, for example transmission windows into the mid-IR, strong nonlinear optical coefficients, light emission, and optical-to-electrical conversion capabilities. Though there have been demonstrations of microstructured optoelectronic fibers [64,65], by far the most common design has been the step-index fiber and much of the research has been focused on integrating new materials and reducing light propagation losses. This section reviews the progress made in this endeavor.

### 3.1 Elemental semiconductors

Silicon and germanium have been the dominant semiconductors of the electronics era and much of the research and development that has contributed to their dominance has been leveraged to establish planar waveguide photonics platforms for each material. Both materials are very well developed and hold great promise for photonics as they have wide transparent windows, 1–7 μm for silicon and 2–15 μm for germanium and high Kerr nonlinear coefficients, which enables nonlinear applications such as supercontinuum generation, four-wave mixing and Raman lasing [10,26,27,66]. It is no surprise then that these materials have been the most widely explored for the semiconductor optoelectronic fiber.

Silicon core fiber has been produced by both the HPCVD and MCD methods, however, the high melting temperature precludes the material from being used in the PAMF deposition process. As-grown silicon fibers produced by the HPCVD are inevitably amorphous or polycrystalline and the high level of absorption and light scattering from the amorphous material and/or grain boundaries is a major source of propagation loss. This means that, to be useful for waveguiding applications, these fibers must undergo some processing to augment the crystal quality. The two-step thermal annealing process has been shown to be effective at reducing the stress-induced defects and promoting larger grain growth and optical transmission losses of 1 dB/cm at wavelength of 2.2 μm (Fig. 3(a)) has been demonstrated [53]. However, it is noted that these losses are very strongly wavelength dependent and at the telecoms transmission wavelength of 1.55 μm, the losses are prohibitively high at 6 dB/cm. One approach to producing low loss fiber with a silicon core is to deposit the material in an amorphous state and to use hydrogen to passivate the strongly absorbing dangling bonds in the material. The approach takes advantage of the low processing temperatures that the HPCVD process allows and this is the only technique that has been able to produce such material. The hydrogen passivation of the core material reduces the fiber's transmission losses from 30 dB/cm to less than 2 dB/cm in the telecoms wavelength region. In contrast, the as-drawn MCD produced silicon fibers can only be produced with crystalline cores and these can have comparatively low transmission losses of 2.7 dB/cm at 1.3 μm [26,45]. However, as the core is reduced in size contaminant in-diffusion from the cladding becomes problematic and increases the transmission losses. Though interface modifiers added in the drawing process can limit the in-diffusion, it is typical that, to produce small core fibers, the fiber must be post-processed, by tapering.

In recent years, there has been a move toward using silicon core fibers for applications in the mid-IR and propagation losses have been reported to be $<$ 2 dB/cm at up to 4 μm and 1.62 dB/cm between 4.7 and 4.9 μm [67,68]. Germanium core fibers also excel in the mid-IR and the first demonstration of was in 2007 by using the HPCVD approach, although, in this study, the optical properties were not measured [69]. The HPCVD process for producing hydrogenated silicon core material was modified to produce germanium fibers, leading to the first germanium optical fibers. Though relatively high loss, with measurements of 17.8 and 4.8 dB/cm at 3.39 and 10.6 μm respectively, this paper represented an important demonstration of waveguiding in such fibers [70]. More recently, amorphous germanium fibers produced via the HPCVD process were successfully recrystallized using a laser and a 9 mm single crystal Ge core fiber was successfully produced. The resultant fiber exhibited a very low propagation loss of 1.33 dB/cm at 2 μm [71].

Initially MCD produced Ge core fibers had high crystallinity but exhibited high transmission losses owing to the diffusion of impurities from the borosilicate glass cladding. However, by switching to the higher softening

**Fig. 3** (a) Cross-section scanning electron micrograph of silicon core fiber (scale bar: 20 μm). Reprinted with permission from Ref. [53]. Copyright 2016, American Chemical Society. (b) Cross-section X-ray computed tomography image of SiGe core fiber after recrystallization (scale bar: 200 μm). Reprinted from Ref. [61]. (c) Scanning electron micrograph of ZnSe core fiber (scale bar: 5 μm). Reprinted with permission from Ref. [78]. Copyright 2011, WILEY-VCH Verlag GmbH & Co. KGaA. (d) Image of the guided optical mode at 1.55 μm of $Cr^{2+}$:ZnSe core fiber. Reprinted with permission from Ref. [79]. Copyright 2020, The Optical Society. (e) Scanning electron micrograph of SeTe core fiber. Reprinted with permission from Ref. [80]. Copyright 2018, Elsevier temperature silica cladding, lower oxygen content Ge cores have been achieved and a low loss of 0.7 dB/cm at 3.39 μm was reported [47,72]. At longer wavelengths, the transmission losses observed can be higher and this is generally accredited to the interaction of the light with the highly absorbing cladding material. However, recent reports have shown that laser annealing can greatly improve the transmission losses, suggesting that it is the core material itself that is causing a large portion of the observed loss. The laser annealing can improve the transmission losses from 6.77 to 2.05 dB/cm at 4.7–4.9 μm [60].

As with silicon, germanium is an excellent material for mid-IR applications and the field of semiconductor optoelectronics fibers has recognized this with a number of recent explorations of these fibers in the mid-IR. For example, 5.82–6.28 μm light has been successfully transmitted through Ge-core/borosilicate clad fibers with losses measured between 3.1 and 9.1 dB/cm. The losses being attributed to the impurity diffusion from the cladding, grain boundary in the core and the evanescent field being absorbed by the cladding [73]. Undoubtedly a high-quality core is crucial for achieving good optical transmission, however, the cladding absorption cannot be ignored. A glass/metal/semiconductor composite fiber was proposed by Shi et al., in which Tin was used to isolate the core and cladding. It is believed that the metallic layer blocks the evanescent field and also reflects the field back to the core. Although transmission was reported, a measured loss of 23.5 dB/cm at 3.39 μm was still relatively high, which was probably related to the crystal quality of the Ge core [74]. After much progress, the field is reaching the conclusion that the cladding materials must be replaced for more significant progress for the technology in the mid-IR.

Though most exploration and experimentation has been in Si and Ge, other core materials have been demonstrated, such as selenium (Se) and tellurium (Te). These materials are attractive as they have high transparency in the mid-IR range (up 20 and 30 μm respectively), high Raman gain, strong nonlinear optical response and photoconductivity [75,76]. Crystalline selenium fiber with phosphate glass cladding has been produced using the MCD method and the propagation loss can be reduced from 2.6 to 1.5 dB/cm at 1.31 μm by employing a two-step annealing process [76,77]. However, it is noted that the Se and Te fibers produced to date have core diameters larger than 50 μm, which is not suitable for single mode transmission and nonlinear applications.

### 3.2 Semiconductor alloys

Semiconductor alloys allow the tuning of material properties via the adjustment of the alloy's composition, for instance, the bandgap, refractive index and nonlinear optical properties can be modified. The silicon-germanium alloy, SiGe, is among the most well-known in the electronics industry and like its elemental counterparts has now been adopted as a planar waveguide platform for mid infrared applications. For example, supercontinuum generation spanning from 3.0 to 8.5 μm has recently been observed from a SiGe on-chip waveguide with a low propagation loss ( < 0.4 dB/cm) [81]. SiGe alloy optoelectronic fiber production has been explored using the MCD method. However, the as-drawn fibers suffer from poor crystal homogeneity and, thus, post-processing is necessary to improve the optical transmission [61,82]. An X-ray computed tomography image of a SiGe core fiber after laser recrystallization is shown in Fig. 3(b). The reported propagation losses of this fiber was 12 and 9.7 dB/cm at 1.55 and 2 μm, respectively, while an HPCVD produced fiber had a loss of 7 dB/cm at 1.55 μm. These losses are relatively high compared to their constituent unary counterparts and further improvement is required; likely facilitated through optimisation of the post-processing parameters. The different solidification temperatures of silicon and germanium allow the composition of the alloy to be locally altered by $CO_2$ laser annealing and Wu et al. used the technique to demonstrate radial and axial Ge concentration variations within a SiGe fiber [62,83]. Compositional variations along the fiber's length is a potential route for the fabricating in-fiber diode-like structure, leading to photodetection and solar cell applications, while the radial graded core structure is particular useful for infrared transmission beyond 4 μm as the Ge-rich region serves as the preferential guiding medium. Numerical studies suggested a graded-index core structure could have a 100-time lower transmission loss in the mid-IR region and also suppress the strong absorption by the silica cladding [84].

ZnSe is particularly attractive for mid-infrared applications because of its wide transmission window, from 0.5 to 22 μm [85]. Moreover, chalcogenide compounds typically possess a second-order nonlinear coefficient because of their noncentrosymmetric crystal structures, which is necessary for second-order nonlinear optical processes, such as second harmonic generation. Using the HPCVD method, polycrystalline ZnSe fiber with low propagation losses of 0.5–0.9 dB/cm from 2 to 2.4 μm wavelength have been demonstrated by Sparks et al. [78]. However, unlike the fabrication of silicon and germanium core fibers, in which completely filled core can be achieved, HPCVD grown ZnSe fiber will typically have a few hundred nanometre diameter pore at its center because the reaction by-product cannot be extracted totally, as shown in Fig. 3(c). This central pore can affect the light coupling, for instance, Gaussian modes cannot be supported. It should be noted that there has been no demonstration of a ZnSe fiber produced via the MCD route as the material has a propensity to sublimate at the required elevated temperatures.

Not only an excellent transmission material for the mid-IR, ZnSe can be doped with transition metal ions and become an efficient gain medium, for instance $Cr^{2+}$:ZnSe and $Fe^{2+}$:ZnSe emit light at 2–3 μm and 3.7–5 μm respectively [86,87]. A homogenous distribution of dopants with a concentration between $10^{18}$ and $10^{19}$ $cm^{-3}$ is desirable in this instance as low concentration results in limited lasing power while excess concentration leads to fluorescence quenching. Diffusion doping is a common strategy to incorporate transition metal ions in bulk ZnSe, however the uniform concentration region can be produced is about 1 mm and the process is not compatible for fabricating fibers. Considering the volatility and stability in high temperature and pressure (the condition for HPCVD) of various organometallic molecules, it was determined that metallocene bis(cyclopentadienyl)chromium(II) ($Cp_2Cr$) and n-butylferrocene are the suitable precursors for Cr and Fe dopant in the HPCVD ZnSe fabrication [79,88]. The mode profile at 1.55 μm of $Cr^{2+}$:ZnSe is shown in Fig. 3(d). This work provides a route to semiconductor optoelectronic fiber-based lasers.

III-V semiconductor alloys are also attractive for materials for optoelectronic fibers often being good light emitters and having good mid/far-IR transparency, for instance, InSb is transparent from 7.3 to 30 μm. However, due to the oxygen and phosphorous contamination from the cladding and free carrier absorption during the MCD production, the high propagation loss of InSb fibers limits its practical operation [89]. On the other hand, emission at 1.7 μm has been observed from polycrystalline GaSb fibers [90]. Extending this work, Song et al. produced fibers with Si cores interspersed with regions of GaSb and used $CO_2$ laser recrystallization to aggregates large regions of GaSb, thus, simultaneously improving crystal quality and increasing the emission intensity to 10 times that of the as-drawn fiber [91]. In addition, a propagation loss of 2 dB/cm at 1.55 μm has been reported for crystalline selenium telluride fibers prepared by MCD with annealing, see Fig. 3(e), and the photoconductivity, sensitivity to light and stress have also been studied [80,92].

## 4 Applications

### 4.1 Nonlinear effects

Nonlinear optics is a well-established field that is now technologically important to many applications, for example, laser technology, communications, quantum optics, and imaging. Many semiconductors have very strong higher order optical nonlinearities and this is an attractive feature of the semiconductor optoelectronic fiber. Though, materials such as ZnSe have the potential to have a second order nonlinearity all of the demonstrations of nonlinear activity in semiconductor optical fibers have been in centrosymmetric crystal systems and the majority in the silicon fiber platform.

#### 4.1.1 Frequency generation

Supercontinuum generation of laser light occurs in a material with a strong third order nonlinearity, such as silicon. It is observed as a large spectral broadening of an input pulse. The broadening itself is a result of a number of nonlinear optical processes such as self-phase modulation and four-wave-mixing occurring simultaneously [93]. The first nonlinear optics experiments undertaken in the semiconductor optoelectronic fiber platform were on the hydrogenated amorphous silicon core fiber. This was the first platform to have low losses and small core size, thus allowing the energy density to be high enough to access the material's nonlinear optical properties. By reducing the core diameter further to (1.7 μm) and accessing the anomalous dispersion regime of the waveguide, an octave spanning spectrum spanning from 1.64 to 3.37 μm was generated from an input wavelength at 2.28 μm, shown in Fig. 4(a). The high figure of merit of amorphous silicon and the low loss of the fiber were vital for such broadband generation [94]. As MCD produced fibers typically have large core diameters, which are not suitable for nonlinear optical applications, Wu et al. used a tapering post-process to reduce the core size of a MCD prepared silicon fiber before demonstrating wavelength conversion and parametric amplification (maximum 9.3 dB gain) at telecom bands based on four-wave mixing [66].

#### 4.1.2 Modulation

A number of mechanisms for the all-optical modulation of light in semiconductor optoelectronic fibers have been demonstrated. The first being the injection of free carriers via optical absorption. The free carrier concentration can alter the refractive index and the absorption of light of the fiber. Exploiting this, Won et al. used a laser to directly excite free carriers in the waveguide and produce all-optical modulation with a frequency of 1.4 MHz with hydrogenated amorphous silicon (a-Si:H) filled fibers [95]. The speed of this process was limited to the recombination time of the free carriers. An extension to this method of absorptive modulation technique is cross-absorption modulation (XAM). Based on non-degenerate two-photon absorption, modulation of a continuous wave signal in a-Si:H core fiber was demonstrated at sub picosecond speeds. However, a slow recovery rate was observed, and this was attributed to the relatively large core increasing the carrier lifetime (87 ns) [96]. A process that relies on the real component of the nonlinear refractive index is cross phase modulation (XPM). In the case of XPM, the signal modulation is achieved by wavelength shifting as a result of large Kerr nonlinearity, instead of absorption. By pumping a-Si:H fiber with a femtosecond laser, Metha et al. demonstrated a blue-shift of 4 nm followed by a red-shift of 10 nm in a probe spectrum centered at 1587 nm, with an extinction ratio of 12 dB, see Figs. 4(b) and 4(c). The speed of such wavelength conversion is about 100 fs, indicating the potential of a-Si:H fiber for ultrafast low power optical modulation [97].

#### 4.1.3 Pulse-shaping

Maintaining the temporal and spectral profiles of a pulsed signal throughout its transmission can be important for signal processing and pulse compression-based technologies. However, this can be difficult for semiconductor photonics technologies as the relatively high losses, when compared to standard optical fibers, alter the extent of the nonlinearity as the pulse propagates through the waveguide. For instance, a soliton requires a fine balance between loss, nonlinearity and dispersion. In semiconductors, one must also contend with other energy loss mechanisms, such as two-photon absorption and free carrier absorption. There have been a number of proposals for pulse shaping in nonlinear semiconductor optoelectronic fibers that employ tapering as a strategy to tune the waveguide properties and compensate for losses. Numerical simulations suggest that soliton formation and propagation can be accommodated by longitudinally decreasing the anomalous dispersion profile over a specific fiber length, as shown in Fig. 4(d) [98]. In contrast, decreasing the normal dispersion profile enables parabolic pulse generation (Fig. 4(e)) and this study also revealed that two-photon absorption and free carrier absorption can cause asymmetry and consequently be used as an alternative mechanism for pulse compression [99].

### 4.2 Optical-to-electrical conversion

At the heart of most semiconductor detection or energy harvesting optoelectronic technologies is the conversion of optical power to electrical power. This is one of the key attractive properties of the semiconductor optoelectronic fiber platform. To this end the platform has been exploited to make in-fiber detectors and "solar threads" [100,101]. It is the prospect of optoelectronic functionality in the core material of the waveguiding fiber that sets this platform apart from other fiber technologies.

The most basic form of light detection is via photoconduction and this process has been demonstrated in germanium optoelectronic fibers. The electric signal can be modulated by increasing the free-carrier density via photo-excitation [102]. Sui et al. demonstrated a photo-response of Ge fiber that operates at speeds up to 100 kHz at 1.55 μm [103]. Lühder et al. demonstrated a photodetector covering the wavelength range 600–1300 nm with a peak sensitivity of 17.8 mV/W at 900 nm. In this instance, the evanescent field from the light propagating through a doped silica core induces charge carriers in a parallel germanium wire, modifying its electrical conductivity. Such an in-fiber device can potentially allow photo-detection without the need of a 'dead-end' detector [104].

Charge extraction can be enhanced by adapting traditional electronic device structures for the fiber geometry.

**Fig. 4** (a) Measured spectra of a supercontinuum generated in a silicon fiber; pump powers labeled in the legend. Dashed lines are a guide to show the power dependent four-wave mixing (FWM) frequency detuning. Reprinted with permission from Ref. [94]. Copyright 2014, The Optical Society. (b) Femtosecond probe spectrogram from XPM using a silicon fiber. Reprinted with permission from Ref. [97]. Copyright 2012, The Optical Society. (c) Measured spectra at the maximum wavelength shifting showing the extinction ratios for the conversion shown in (b). Reprinted with permission from Ref. [97]. Copyright 2012, The Optical Society. (d) Normalized profiles of soliton evolution in a 10 mm tapered silicon fiber. Reprinted with permission from Ref. [98]. Copyright 2010, The Optical Society. (e) Pulse evolution towards the parabolic regime. Reprinted with permission from Ref. [99]. Copyright 2010, The Optical Society

For example, a coaxial Pt/n-Si Schottky junction, shown in Fig. 5(a), was fabricated via the HPCVD technique and the device had a photo-responsivity with 3 GHz bandwidth, see Fig. 5(b). However, the response at 1550 nm was weaker than at 1310 nm because of the relatively high Schottky barrier, which could be reduced by using metals with lower workfunction or forming a heterojunction with other semiconductors.

A further development demonstrated a p-i-n junction for which the photoresponsivity of the silicon p-i-n fiber (0.3 A/W) is much higher than that of the Schottky junction (1.4 mA/W) with a similar bandwidth and response time. This device, shown in Fig. 5(c), also demonstrated photovoltaic functionality, although just a 0.5% efficiency was reported. It was suggested that a thinner $n^+$ layer and thicker i layer could improve the conversion efficiency while forming a photovoltaic fiber array could enhance the collection efficiency [101].

Finally, strain engineering of the core materials of the semiconductor optoelectronic fibers can be induced and "locked-in" by cladding-core thermal expansion coefficients and, as mentioned earlier, this can be controlled via laser post-processing. Healy et al. revealed that the stress in a silicon core can be tuned by adjusting the laser irradiation time. A bandgap reduction of silicon from 1.11 to 0.59 eV was demonstrated, which allows optical detection up to 2100 nm, as shown in Fig. 5(d) [105].

### 4.3 Terahertz waveguiding and modulation

Low loss waveguiding of terahertz radiation (THz) is of key importance for applications, such as medical imaging, environmental monitoring and security [106]. It has long been recognized that high resistivity silicon is one candidate for THz transmission owing to its low dispersion and absorption in this regime [107]. Simulations proposing propagation of THz light through a fiber with a high resistivity silicon core were presented in 2013 [108], however, an experimental demonstration did not occur until recently [109]. A transmission loss of approximately 4 dB/cm between 2 and 6 THz was reported for a silicon core fiber produced using the MCD method and recrystallized using a $CO_2$ laser. In follow up work, optical modulation of THz signals was demonstrated via free

**Fig. 5** (a) Electrodes fabricated on the Pt/n-Si diode using a focused ion beam system, with platinum electrodes contacting the platinum and $n^+$-Si layers (scale bar: 5 μm). Reprinted from Ref. [100]. (b) Photodetection response of a Pt–Si diode to 10 ps laser pulses at wavelengths of 1310 and 1550 nm, measured by an oscilloscope. Reprinted from Ref. [100]. (c) Scanning electron micrograph of a deposited and cleaved junction in-fiber p-i-n structure. Reprinted with permission from Ref. [101]. Copyright 2013, WILEY-VCH Verlag GmbH & Co. KGaA. (d) Photoconduction of laser-crystallized silicon optical fibers measured as a function of excitation energy (F1: fiber irradiated for a duration of 500 μs, F2: fiber irradiated for a duration of 5 ms) and of a single-crystal standard reference. Reprinted from Ref. [105]

carrier absorption. A maximum modulation depth of 96% was observed. A gold dopant served to reduce the free carrier lifetime from the ms to the ns scale [110].

### 4.4 Lasers

Fiber lasers offer a number of advantages over their counterparts, for example, enhanced gain via longer light-matter interaction lengths, higher coupling efficiency, and high and stable beam quality. However, there are some limitations as, in general, fiber lasers are constructed from rare-earth doped glasses. It is typical that the glass is silica which is a poor host for the dopants and has a narrow transparency window. This limits the operating power due to multiphoton quenching and nonradiative decay [10,78,88,111]. Semiconductor optoelectronic fibers offer a promising alternative to the traditional fiber laser platform as the host materials can be varied drastically allowing access to new wavelength regions and shorter devices. To date there have been few demonstrations of a fiber laser in this platform. ZnSe is known to be a good host for transition metal ions that facilitate light emission. For example, $Cr^{2+}$:ZnSe and $Fe^{2+}$:ZnSe lasers that emit light at 2–3 μm and 3.7–5 μm respectively have been demonstrated [86,87]. Recent work has shown that the HPCVD process for fabricating semiconductor optoelectronic fibers can be modified to produce ZnSe fibers with doped cores. The first demonstration was a pulsed $Cr^{2+}$:ZnSe fiber laser pumped at a wavelength of 2.09 μm that exhibited lasing at 2.3 μm with a threshold about 800 μJ (Fig. 6(a)) [79]. A second demonstration was a continuous wave laser based on a $Fe^{2+}$ doped ZnSe fiber pumped at 2.94 μm, see Fig. 6(b). Lasing at 4.12 μm was observed in this fiber, however, cryogenic temperatures were required owing to short radiative lifetimes and a low slope efficiency of 0.1% was reported [88]. Though, in their infancy and far behind the state-of-the-art for silica-based fiber lasers, these demonstrations clearly show the potential of the semiconductor optoelectronic platform for next generation fiber laser technology.

### 4.5 Multimaterial functional fibers

An exciting advancement in the field of semiconductor optoelectronic fibers is the introduction of more complex in-fiber device structures that are enabled by the innovation

**Fig. 6** (a) Spectral emission of $Cr^{2+}$:ZnSe fiber laser above and below the laser threshold. Reprinted with permission from Ref. [79]. Copyright 2020, The Optical Society. (b) Lasing spectrum of free running $Fe^{2+}$:ZnSe optical fiber at full pump power (600 mW) with cryogenic cooling. Reprinted with permission from Ref. [88]. Copyright 2020, The Optical Society of multimaterial fiber. The fabrication utilizes a similar technique as molten core drawing but instead of using just a core and cladding, various materials are incorporated in the preform in a designed device-like layout. The drawing process 'scales' the design down into a microstructured fiber that can have meter long lengths. An example of the process can be seen in Figs. 7(a) and 7(b) [30].

An early example of this multimaterial semiconduct or optoelectronic fiber technology produced a fiber with a photoconductive response. Bayindir et al. fabricated a structure with a chalcogenide core with metal contacts and a cylindrical-shell resonant cavity surrounding this. The photoconductive response was generated when the illumination wavelength matched that of the resonant wavelength of the cavity. A grid structure formed by the fiber demonstrated the capability of locating the illumination point and also the possibility of identifying the direction of the light [32]. A breakthrough for this technology was the introduction of crystalline semiconductors as this reduced the resistivity of the fibers by five orders of magnitude. Danto et al. used *in situ* and post-drawing crystallization processes to produce an in-fiber p-type chalcogenide semiconductor a thin film with source and drain electrodes (Fig. 7(c)). One additional benefit of the in-fiber thin film structure is that the dark current can be suppressed compared to a solid core structure [112]. This work opened significant opportunities for incorporating higher functionality in functional fibers and fabrics. Similar to the more traditional semiconductor optoelectronic fibers, multimaterial functional fibers can suffer from a degradation of material quality during the drawing process. However, it has been shown that the laser post-processing technique can be adapted to significantly increase the material properties in some instances. For example, one study compared regular annealing methods to laser processing methods for selenium core material [113]. It was shown that the laser-based approach produced a much more ordered material when compared to the traditional

**Fig. 7** (a) Schematic of the fabrication of multimaterial preforms. Reprinted with permission from Ref. [30]. Copyright 2019, WILEY-VCH Verlag GmbH & Co. KGaA. (b) Schematic of the thermal drawing of multimaterial fibers with several embedded materials and functionalities. Reprinted with permission from Ref. [30]. Copyright 2019, WILEY-VCH Verlag GmbH & Co. KGaA. (c) Cross-section scanning electron micrograph of the fiber field-effect device (lower panel) and magnification of one of the two devices (upper panel). Reprinted with permission from Ref. [112]. Copyright 2010, WILEY-VCH Verlag GmbH & Co. KGaA

approach. In this instance, the optoelectronic properties were far superior with orders of magnitude higher responsivity and sensitivity being observed.

One challenge of fabricating multimaterial fiber is maintaining the interface between the semiconductors and the electrodes and a key solution to this was to use a selective breakup technique as developed by Wei et al. [114]. This process is based on the capillary breakup process used to create spheres from the core of semiconductor core fibers, as demonstrated by Gumennik et al. [115]. The selective breakup technique leverages capillary breakup to convert the as-drawn continuous germanium core into spheres that form a bridge between two in-fiber electrodes. This method allows multiple devices integrated in one fiber while each metal-semiconductor-metal device exhibits a photoresponse. Another innovative approach to creating contacts between the electrodes and the in-fiber device is to feed the electrical conductors into hollow channels that flank the devices during the drawing process. In this solution, the electrodes are not drawn but lateral separation of these wires is reduced as the fiber is necked-down until intimate electrical contact is made with the devices. This allows for very low drawing temperatures and fully formed devices such as diodes to be included in the process; both light emitting and detecting devices have been demonstrated using this technique [116].

An exciting recent development in the multimaterial fiber platform is the introduction of micro- and nano-structured materials. Nano-materials offer an additional degree of freedom of design for the optoelectronic fibers. For example, nanowires can have tunable optoelectronic properties and strike an efficient balance between excellent light absorption and charge extraction for optoelectronic processes. Yan et al. demonstrated the integration of selenium nanowires into the fiber geometry [117]. The team used a sonochemical synthesis technique developed for the as-drawn fibers and the nanowires were not only grown at the exposed fiber cross-section but also along their length. This approach yielded photoconducting devices with high responsivity and sensitivity, and fast response. This is an important advancement in the field that could inform the next generation of multimaterial optoelectronic fiber technologies.

## 5 Conclusion

Thanks to the advances in fabrication and post-processing techniques, high quality semiconductor core fibers have been realized and the technology is now being exploited for impactful applications. While a wide range of materials have been demonstrated as amenable to fiber fabrication via the HPCVD, PAMF, and MCD techniques, the majority of the functional demonstrations have been attempted on silicon or germanium core fibers. The semiconductor core fibers not only extend the transmission range of optical fiber to the mid-infrared region but also broaden its uses in various applications. For example, the early demonstrations of mid-IR supercontinuum generation and the semiconductor optoelectronic fiber laser are stepping stones for applications in mid-infrared sensing, spectroscopy, and medical applications. It is anticipated that the semiconductor fiber platform will be important in a wide array of technological areas, from quantum optics, through telecommunication, to wearable electronics.

**Acknowledgements** The authors acknowledge UKRI and EPSRC for financial support under the grant EP/R021503/1.

**Open Access** This article is licensed under a Creative Commons Attribution 4.0 International License, which permits use, sharing, adaptation, distribution and reproduction in any medium or format, as long as you give appropriate credit to the original author(s) and the source, provide a link to the Creative Commons licence, and indicate if changes were made. The images or other third party material in this article are included in the article's Creative Commons licence, unless indicated otherwise in a credit line to the material. If material is not included in the article's Creative Commons licence and your intended use is not permitted by statutory regulation or exceeds the permitted use, you will need to obtain permission directly from the copyright holder.

To view a copy of this licence, visit http://creativecommons.org/licenses/by/4.0/.
